# Crystal structure of bis­[4′-(1,4,7,10-tetra­oxa-13- aza­cyclo­penta­decan-13-yl)-2,2′:6′,2′′-terpyridine]­cobalt(III) tris­(perchlorate) methanol monosolvate monohydrate

**DOI:** 10.1107/S2056989015014164

**Published:** 2015-07-31

**Authors:** Hitomi Ohmagari, Manabu Nakaya, Ryo Ohtani, Masaaki Nakamura, Shinya Hayami

**Affiliations:** a Department of Chemistry, Graduate School of Science and Technology, Kumamoto University, 2-39-1 Kurokami Kumamoto 860-8555, Japan

**Keywords:** crystal structure, terpyridine, crown ether, hydrogen bonding, C—H⋯π inter­actions, π–π stacking inter­actions

## Abstract

In the title compound, [Co(C_25_H_30_N_4_O_4_)_2_](ClO_4_)_3_·CH_3_OH·H_2_O, the metal atom is coordinated by two tridentate crown ether terpyridine ligands, forming a distorted CoN_6_ octa­hedron. The three pyridine rings in each crown–terpyridine ligand are approximately coplanar [maximum deviations = 0.088 (12) and 0.102 (15) Å] and the mean planes through the three pyridine rings are perpendicular to each other, making a dihedral angle of 89.95 (17)°. An intra­molecular C—H⋯π inter­action is observed between the two terpyridine ligands. In the crystal, O—H⋯O and C—H⋯O hydrogen bonds, a π–π stacking inter­action [centroid–centroid distance = 3.923 (7) Å] and a C—H⋯π inter­action connect the complex cation, the perchlorate anions and the two types of solvent molecules, forming a three-dimensional network.

## Abstract 2

The title compound, bis­[4′-(1,4,7,10-tetra­oxa-13-aza­cyclo­penta­decan-13-yl)-2,2′:6′,2"-terpyridine]­cobalt(III) tris­(perchlorate) methanol monosolvate monohydrate, is a novel complex in which the metal centre is hexa­coordinated.

## Chemical context   

Metal complexes with terpyridine derivatives, [*M*(*R*-terpy)]*X*_2_ (*M* = transition metal ions; *R*-terpy = substituted 2,2′:6′,2′′-terpyridine; *X* = anion), have been investigated because of their inter­esting properties such as magnetic and photochemical characteristics. Cobalt(II) complexes with *R*-terpy ligands are known as spin-crossover compounds. Previously, we observed the unique spin-transition behavior in [Co(II)(*R*-terpy)_2_](BF_4_)_2_ with long-alkyl­ated terpyridine ligands, and showed that the magnetic behaviors are influenced not only by inter-chain inter­actions between long alkyl chains but also by π–π stacking inter­actions between terpyridine moieties (Hayami *et al.*, 2011 ▸). We suggested that inter­molecular inter­actions play an important role for the magnetic behaviors of metal complexes. Herein we focused on the terpyridine ligand with a crown ether ring, and synthesized the title compound.
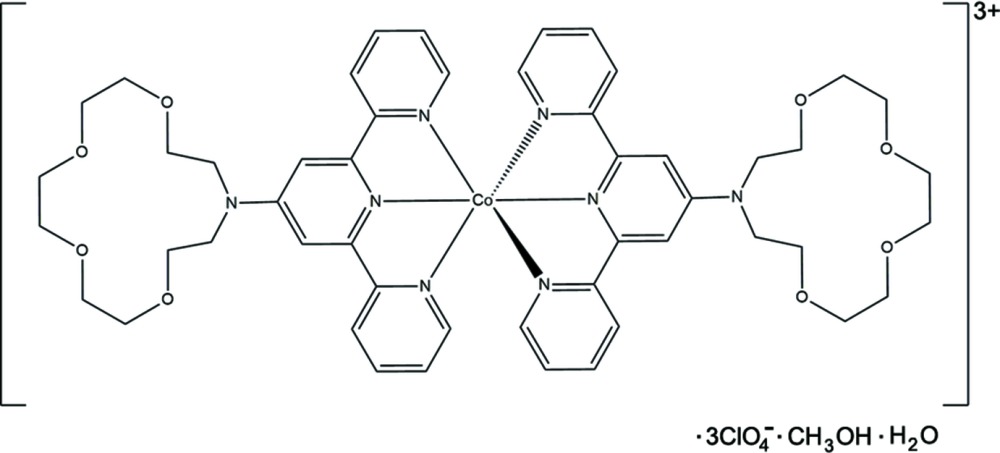


## Structural commentary   

**Figure 1:**
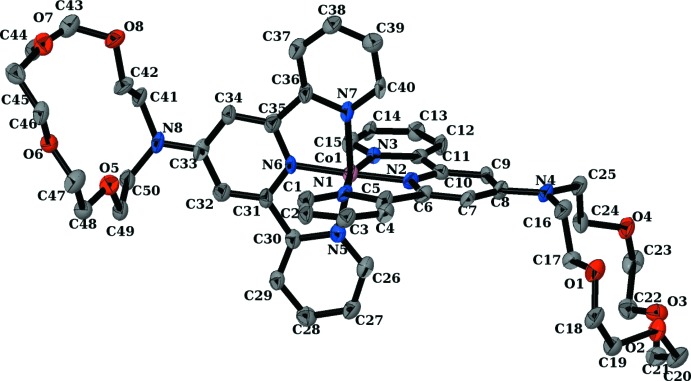
A view of the complex cation of the title compound, showing displacement ellipsoids at the 50% probability level.

The asymmetric unit of the title compound consists of one [Co(crown–terpy)_2_] complex cation, three perchlorate anions, one methanol solvent mol­ecule and one water solvent mol­ecule. The mol­ecular structure of the complex cation is shown in Fig. 1 ▸. The cobalt(III) atom is octa­hedrally coordinated by six nitro­gen atoms of two crown–terpy ligands, *i.e.* an N_6_ donor set. The coordination bond lengths are typical of those for low-spin cobalt(III) compounds. The Co—N distances of the central pyridine in the terpyridine unit [1.844 (9) Å] are shorter than the Co—N distances of the side pyridine in the terpyridine unit [1.910 (10)–1.949 (10) Å], which induces a pronounced distortion of the CoN_6_ octa­hedron. The three pyridine rings in each crown–terpy ligand are approximately coplanar [maximum deviations 0.102 (15) and 0.088 (12) Å], and the two mean planes through the three pyridine rings in the complex are nearly perpendicular to each other, making a dihedral angle of 89.95 (17)°.

## Supra­molecular feature   

**Figure 2:**
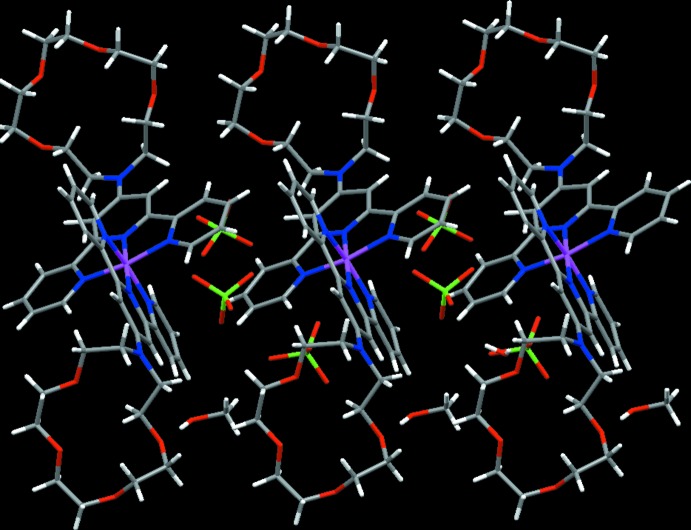
Crystal packing diagram of the title compound.

The overall packing of structure is shown in Fig. 2 ▸. In the crystal, O—H⋯O hydrogen bonds are formed between the water mol­ecule and the complex cation, between the water mol­ecule and the perchlorate anion, and between the methanol mol­ecule and the complex cation (Table 1 ▸). Tog­ether with these hydrogen bonds, C—H⋯O hydrogen bonds connect the four components, forming a three-dimensional network. In addition, between pyridine rings of neighboring mol­ecules a C—H⋯π inter­action (Table 1 ▸) and a π–π inter­action are observed. The centroid–centroid distance between the N1/C1–C5 and N3/C11–C15 pyridine rings is 3.923 (7) Å.

## Synthesis and crystallization   

The crown-terpyridine ligand was prepared by the reaction of 4′-bromo-2,2′:6′,2′′-terpyridine (249.2 mg, 1 mmol) and 1,4,7,10-tetra­oxa-13-aza­cyclo­penta­decane (459.2 mg, 1 mmol) in DMF. The mixed solution was evaporated to give the ligand as a white powder. Co(ClO_4_)_3_ (68.16 mg, 0.5 mmol) dissolved in methanol (20 ml) was poured dropwise into a solution of the crown–terpy ligand (100 mg, 0.21 mmol) in 1:1 methanol–chloro­form. The precipitate formed immediately and was filtered. Single crystals of the title compound suitable for X-ray diffraction were obtained from a methanol solution.

## Refinement   

Crystal data, data collection and structure refinement details are summarized in Table 2 ▸. H atoms in the complex cation and the methanol mol­ecule were placed in calculated positions (C—H = 0.93–0.97 Å and O—H = 0.82 Å) and allowed to ride on their parent atoms with *U*_iso_(H) = 1.2*U*_eq_(C) and 1.5*U*_eq_(O, C_meth­yl_). The positions of the H atoms of the water mol­ecule were refined with restraints of O—H = 0.85 (2) and H⋯H = 1.38 (2) Å, and with *U*_iso_(H) = 1.5*U*_eq_(O).

## Supplementary Material

Crystal structure: contains datablock(s) global, I. DOI: 10.1107/S2056989015014164/is5400sup1.cif

Structure factors: contains datablock(s) I. DOI: 10.1107/S2056989015014164/is5400Isup2.hkl

CCDC reference: 1415327

Additional supporting information:  crystallographic information; 3D view; checkCIF report

## Figures and Tables

**Table 1:** Hydrogen-bond geometry (, ) *Cg*1 and *Cg*2 are the centroids of the N7/C36C40 and N2/C6C10 pyridine rings, respectively.

*D*H*A*	*D*H	H*A*	*D* *A*	*D*H*A*
O21H21O8	0.82	1.99	2.802(15)	173
O22H5O9	0.86(13)	2.11(14)	2.960(19)	172(15)
O22H6O5	0.86(12)	2.17(11)	2.934(13)	148(14)
C4H4O18	0.93	2.56	3.299(16)	137
C15H15O11^i^	0.93	2.54	3.35(2)	145
C16H16*B*O6^ii^	0.97	2.58	3.491(15)	157
C20H20*B*O10^ii^	0.97	2.54	3.50(2)	170
C22H22*A*O13^iii^	0.97	2.41	3.365(17)	168
C25H25*A*O6^ii^	0.97	2.59	3.517(14)	160
C25H25*B*O20^i^	0.97	2.49	3.331(15)	145
C26H26O16^iii^	0.93	2.54	3.400(17)	155
C27H27O14^iii^	0.93	2.45	3.249(16)	143
C28H28O22^iii^	0.93	2.47	3.290(17)	147
C29H29O7^iii^	0.93	2.51	3.437(14)	175
C37H37O21	0.93	2.35	3.276(16)	177
C44H44*A*O20^iv^	0.97	2.49	3.297(17)	141
C3H3*Cg*1^v^	0.93	2.70	3.540(15)	151
C40H40*Cg*2	0.93	2.97	3.713(12)	138

Symmetry codes: (i) 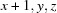; (ii) 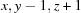; (iii) 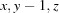; (iv) 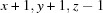; (v) 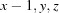.

**Table 2:** Experimental details

Crystal data	
Chemical formula	[Co(C_25_H_30_N_4_O_4_)_2_](ClO_4_)_3_CH_4_OH_2_O
*M*_r_	1308.42
Crystal system, space group	Triclinic, *P*1
Temperature (K)	100
*a*, *b*, *c* ()	8.8080(8), 9.5032(8), 16.9321(14)
, , ()	84.237(2), 81.674(2), 85.652(3)
*V* (^3^)	1392.6(2)
*Z*	1
Radiation type	Mo *K*
(mm^1^)	0.54
Crystal size (mm)	0.50 0.10 0.05

Data collection	
Diffractometer	Rigaku R-AXIS RAPID
Absorption correction	Multi-scan (*ABSCOR*; Rigaku, 1995 ▸)
*T*_min_, *T*_max_	0.370, 0.973
No. of measured, independent and observed [*F* ^2^ > 2(*F* ^2^)] reflections	13693, 10375, 6395
*R*_int_	0.098
(sin /)_max_ (^1^)	0.649

Refinement	
*R*[*F* ^2^ > 2(*F* ^2^)], *wR*(*F* ^2^), *S*	0.086, 0.256, 1.04
No. of reflections	10375
No. of parameters	774
No. of restraints	6
H-atom treatment	H atoms treated by a mixture of independent and constrained refinement
_max_, _min_ (e ^3^)	0.96, 1.09
Absolute structure	Flack *x* determined using 1715 quotients [(*I* ^+^)(*I* )]/[(*I* ^+^)+(*I* )] (Parsons Flack, 2004 ▸)
Absolute structure parameter	0.02(3)

Computer programs: *RAPID-AUTO* (Rigaku, 1995 ▸), *SIR2004* (Burla *et al.*, 2005 ▸), *SHELXL2014* (Sheldrick, 2015 ▸), *CrystalStructure* (Rigaku, 2014 ▸).

## supplementary crystallographic information

### Crystal data

**Table:**

[Co(C_25_H_30_N_4_O_4_)_2_](ClO_4_)_3_·CH_4_O·H_2_O	*Z* = 1
*M**_r_* = 1308.42	*F*(000) = 682.00
Triclinic, *P*1	*D*_x_ = 1.560 Mg m^−^^3^
*a* = 8.8080 (8) Å	Mo *K*α radiation, λ = 0.71075 Å
*b* = 9.5032 (8) Å	Cell parameters from 9503 reflections
*c* = 16.9321 (14) Å	θ = 3.1–27.5°
α = 84.237 (2)°	µ = 0.54 mm^−^^1^
β = 81.674 (2)°	*T* = 100 K
γ = 85.652 (3)°	Platelet, brown
*V* = 1392.6 (2) Å^3^	0.50 × 0.10 × 0.05 mm

### Data collection

**Table:**

Rigaku R-AXIS RAPID diffractometer	6395 reflections with *F*^2^ > 2σ(*F*^2^)
Detector resolution: 10.000 pixels mm^-1^	*R*_int_ = 0.098
ω scans	θ_max_ = 27.5°
Absorption correction: multi-scan (*ABSCOR*; Rigaku, 1995)	*h* = −11→11
*T*_min_ = 0.370, *T*_max_ = 0.973	*k* = −12→12
13693 measured reflections	*l* = −21→21
10375 independent reflections	

### Refinement

**Table:**

Refinement on *F*^2^	Hydrogen site location: inferred from neighbouring sites
*R*[*F*^2^ > 2σ(*F*^2^)] = 0.086	H atoms treated by a mixture of independent and constrained refinement
*wR*(*F*^2^) = 0.256	*w* = 1/[σ^2^(*F*_o_^2^) + (0.1411*P*)^2^] where *P* = (*F*_o_^2^ + 2*F*_c_^2^)/3
*S* = 1.03	(Δ/σ)_max_ < 0.001
10375 reflections	Δρ_max_ = 0.96 e Å^−^^3^
774 parameters	Δρ_min_ = −1.09 e Å^−^^3^
6 restraints	Absolute structure: Flack *x* determined using 1715 quotients [(*I*^+^)-(*I*^-^)]/[(*I*^+^)+(*I*^-^)] (Parsons & Flack, 2004)
Primary atom site location: structure-invariant direct methods	Absolute structure parameter: 0.02 (3)
Secondary atom site location: difference Fourier map	

### Special details

**Table:**

Geometry. ENTER SPECIAL DETAILS OF THE MOLECULAR GEOMETRY
Refinement. Refinement was performed using all reflections. The weighted *R*-factor (*wR*) and goodness of fit (*S*) are based on *F*^2^. *R*-factor (gt) are based on *F*. The threshold expression of *F*^2^ > 2.0 σ(*F*^2^) is used only for calculating *R*-factor (gt).

### Fractional atomic coordinates and isotropic or equivalent isotropic displacement parameters (Å^2^)

**Table:**

	*x*	*y*	*z*	*U*_iso_*/*U*_eq_	
Co1	0.76909 (15)	0.08790 (13)	0.13275 (9)	0.0288 (4)	
Cl1	0.3213 (4)	0.1579 (4)	−0.1139 (2)	0.0467 (8)	
Cl2	0.4623 (5)	0.5336 (3)	0.2794 (2)	0.0496 (9)	
Cl3	0.0656 (4)	0.0376 (3)	0.43350 (17)	0.0349 (7)	
O1	0.2434 (10)	−0.2957 (9)	0.5627 (5)	0.040 (2)	
O2	0.2493 (11)	−0.5893 (9)	0.6131 (5)	0.039 (2)	
O3	0.5560 (11)	−0.7130 (9)	0.5627 (5)	0.040 (2)	
O4	0.7922 (11)	−0.5239 (8)	0.5341 (5)	0.038 (2)	
O5	0.6674 (11)	0.4750 (8)	−0.2506 (5)	0.041 (2)	
O6	0.5788 (10)	0.7563 (9)	−0.2955 (5)	0.0355 (19)	
O7	0.7867 (12)	0.9733 (9)	−0.2658 (5)	0.043 (2)	
O8	0.9854 (12)	0.7656 (9)	−0.1865 (6)	0.044 (2)	
O9	0.2713 (15)	0.2838 (16)	−0.0750 (8)	0.089 (5)	
O10	0.379 (2)	0.1901 (16)	−0.1895 (8)	0.119 (7)	
O11	0.1913 (16)	0.0748 (15)	−0.1079 (11)	0.101 (5)	
O12	0.433 (2)	0.0815 (19)	−0.0708 (14)	0.147 (8)	
O13	0.4992 (13)	0.4261 (11)	0.3397 (6)	0.059 (3)	
O14	0.5172 (15)	0.4906 (12)	0.2014 (5)	0.061 (3)	
O15	0.2926 (16)	0.5578 (13)	0.2866 (9)	0.090 (5)	
O16	0.532 (2)	0.6637 (12)	0.2864 (7)	0.092 (5)	
O17	0.1782 (13)	0.1141 (11)	0.4618 (6)	0.056 (3)	
O18	0.0058 (11)	0.1245 (9)	0.3700 (6)	0.047 (2)	
O19	0.1408 (12)	−0.0920 (10)	0.4047 (6)	0.049 (2)	
O20	−0.0586 (11)	0.0040 (10)	0.4955 (6)	0.050 (3)	
O21	1.0569 (13)	0.7203 (12)	−0.0296 (7)	0.058 (3)	
O22	0.5440 (14)	0.4499 (11)	−0.0796 (6)	0.058 (3)	
N1	0.5582 (12)	0.1554 (9)	0.1505 (5)	0.029 (2)	
N2	0.7229 (11)	0.0005 (10)	0.2348 (6)	0.027 (2)	
N3	0.9705 (12)	−0.0059 (10)	0.1438 (5)	0.028 (2)	
N4	0.6214 (12)	−0.1840 (10)	0.4670 (6)	0.031 (2)	
N5	0.7192 (12)	−0.0609 (10)	0.0721 (6)	0.030 (2)	
N6	0.8196 (12)	0.1781 (10)	0.0318 (5)	0.030 (2)	
N7	0.8311 (12)	0.2623 (10)	0.1661 (5)	0.029 (2)	
N8	0.9469 (13)	0.3779 (10)	−0.1943 (6)	0.034 (2)	
C1	0.4792 (16)	0.2376 (12)	0.0982 (7)	0.035 (3)	
C2	0.3246 (16)	0.2856 (12)	0.1191 (7)	0.038 (3)	
C3	0.2545 (16)	0.2508 (13)	0.1965 (8)	0.037 (3)	
C4	0.3316 (15)	0.1623 (12)	0.2487 (7)	0.034 (3)	
C5	0.4812 (13)	0.1165 (11)	0.2256 (7)	0.028 (2)	
C6	0.5781 (14)	0.0217 (11)	0.2748 (7)	0.029 (3)	
C7	0.5383 (15)	−0.0367 (12)	0.3520 (7)	0.032 (3)	
C8	0.6496 (14)	−0.1234 (12)	0.3894 (7)	0.028 (3)	
C9	0.8020 (13)	−0.1426 (11)	0.3471 (6)	0.027 (2)	
C10	0.8303 (14)	−0.0796 (12)	0.2689 (7)	0.029 (2)	
C11	0.9784 (15)	−0.0824 (12)	0.2149 (7)	0.032 (3)	
C12	1.1133 (15)	−0.1560 (11)	0.2315 (7)	0.032 (3)	
C13	1.2438 (15)	−0.1510 (12)	0.1753 (7)	0.034 (3)	
C14	1.2326 (15)	−0.0707 (13)	0.1017 (7)	0.034 (3)	
C15	1.0963 (16)	−0.0005 (12)	0.0896 (7)	0.037 (3)	
C16	0.4678 (14)	−0.1757 (12)	0.5139 (7)	0.032 (3)	
C17	0.3766 (15)	−0.3032 (13)	0.5053 (8)	0.036 (3)	
C18	0.1299 (15)	−0.3849 (13)	0.5462 (8)	0.040 (3)	
C19	0.1884 (17)	−0.5405 (14)	0.5399 (8)	0.042 (3)	
C20	0.2917 (17)	−0.7374 (14)	0.6149 (9)	0.045 (3)	
C21	0.4214 (16)	−0.7781 (13)	0.5537 (8)	0.040 (3)	
C22	0.6712 (17)	−0.7252 (13)	0.4948 (7)	0.040 (3)	
C23	0.8174 (17)	−0.6641 (13)	0.5112 (8)	0.043 (3)	
C24	0.7705 (17)	−0.4219 (12)	0.4687 (7)	0.037 (3)	
C25	0.7350 (15)	−0.2780 (11)	0.5036 (7)	0.033 (3)	
C26	0.6618 (14)	−0.1841 (12)	0.1009 (7)	0.034 (3)	
C27	0.6197 (15)	−0.2781 (13)	0.0522 (7)	0.036 (3)	
C28	0.6395 (17)	−0.2449 (13)	−0.0316 (8)	0.038 (3)	
C29	0.7026 (15)	−0.1173 (12)	−0.0633 (7)	0.034 (3)	
C30	0.7400 (13)	−0.0264 (11)	−0.0100 (6)	0.027 (2)	
C31	0.8041 (15)	0.1133 (12)	−0.0339 (7)	0.033 (3)	
C32	0.8445 (14)	0.1755 (12)	−0.1095 (7)	0.033 (3)	
C33	0.9060 (15)	0.3130 (13)	−0.1208 (7)	0.033 (3)	
C34	0.9234 (14)	0.3770 (12)	−0.0500 (6)	0.031 (3)	
C35	0.8782 (14)	0.3093 (12)	0.0243 (7)	0.033 (3)	
C36	0.8802 (14)	0.3583 (11)	0.1029 (7)	0.031 (3)	
C37	0.9269 (15)	0.4901 (12)	0.1171 (7)	0.036 (3)	
C38	0.9210 (17)	0.5219 (13)	0.1950 (8)	0.043 (3)	
C39	0.8734 (17)	0.4287 (14)	0.2562 (8)	0.041 (3)	
C40	0.8241 (15)	0.2973 (12)	0.2417 (6)	0.031 (3)	
C41	1.0265 (17)	0.5099 (12)	−0.2036 (7)	0.036 (3)	
C42	0.9121 (16)	0.6387 (11)	−0.1958 (8)	0.040 (3)	
C43	1.0336 (17)	0.8480 (14)	−0.2601 (8)	0.045 (3)	
C44	0.9077 (17)	0.8978 (14)	−0.3117 (8)	0.043 (3)	
C45	0.6521 (18)	0.9962 (14)	−0.3080 (9)	0.045 (3)	
C46	0.5347 (16)	0.8952 (12)	−0.2743 (7)	0.036 (3)	
C47	0.4694 (17)	0.6548 (14)	−0.2622 (9)	0.045 (3)	
C48	0.5297 (17)	0.5122 (14)	−0.2852 (8)	0.043 (3)	
C49	0.7317 (16)	0.3359 (12)	−0.2671 (7)	0.039 (3)	
C50	0.9005 (17)	0.3271 (13)	−0.2658 (7)	0.040 (3)	
C51	1.2209 (19)	0.689 (2)	−0.0377 (11)	0.069 (5)	
H1	0.52847	0.26304	0.04691	0.0416*	
H2	0.27099	0.33921	0.08191	0.0452*	
H3	0.15536	0.28732	0.21306	0.0444*	
H4	0.28238	0.13362	0.29951	0.0406*	
H5	0.460 (11)	0.409 (19)	−0.076 (10)	0.0869*	
H6	0.600 (16)	0.427 (19)	−0.123 (7)	0.0869*	
H7	0.43993	−0.01952	0.37897	0.0387*	
H9	0.87922	−0.19533	0.37099	0.0324*	
H12	1.11622	−0.209	0.2806	0.0388*	
H13	1.33574	−0.19894	0.18579	0.0408*	
H14	1.31708	−0.06576	0.06186	0.0414*	
H15	1.09069	0.05389	0.04119	0.0441*	
H16A	0.41193	−0.0893	0.49601	0.0384*	
H16B	0.47775	−0.17185	0.5699	0.0384*	
H17A	0.34885	−0.29899	0.45173	0.0433*	
H17B	0.43728	−0.39104	0.51536	0.0433*	
H18A	0.09371	−0.34791	0.49631	0.0484*	
H18B	0.04296	−0.38105	0.58843	0.0484*	
H19A	0.10483	−0.59723	0.53314	0.0499*	
H19B	0.26787	−0.54819	0.49415	0.0499*	
H20A	0.20282	−0.78667	0.60781	0.0536*	
H20B	0.31881	−0.7699	0.66753	0.0536*	
H21A	0.44077	−0.88019	0.55882	0.0484*	
H21B	0.39411	−0.75	0.50068	0.0484*	
H21	1.03052	0.73952	−0.07417	0.0872*	
H22A	0.63565	−0.67401	0.4477	0.0482*	
H22B	0.69184	−0.824	0.4847	0.0482*	
H23A	0.85969	−0.72429	0.55344	0.0513*	
H23B	0.89263	−0.66473	0.46331	0.0513*	
H24A	0.68576	−0.44509	0.44261	0.0445*	
H24B	0.86261	−0.41941	0.42964	0.0445*	
H25A	0.69899	−0.29483	0.56047	0.0397*	
H25B	0.82982	−0.23003	0.49808	0.0397*	
H26	0.64963	−0.20782	0.15597	0.0411*	
H27	0.57841	−0.36271	0.07453	0.0430*	
H28	0.61109	−0.30695	−0.06523	0.0458*	
H29	0.71936	−0.09348	−0.11837	0.0404*	
H32	0.83213	0.12896	−0.1537	0.0392*	
H34	0.96538	0.46477	−0.05424	0.0377*	
H37	0.96122	0.55491	0.0746	0.0431*	
H38	0.95067	0.60943	0.2051	0.0518*	
H39	0.87272	0.45066	0.30853	0.0495*	
H40	0.78657	0.23442	0.28449	0.0368*	
H41A	1.0891	0.51881	−0.2557	0.0437*	
H41B	1.09422	0.50689	−0.16298	0.0437*	
H42A	0.8606	0.65387	−0.24308	0.0475*	
H42B	0.83472	0.61967	−0.14974	0.0475*	
H43A	1.11253	0.7924	−0.29157	0.0539*	
H43B	1.08039	0.93084	−0.24759	0.0539*	
H44A	0.94991	0.95862	−0.35793	0.0514*	
H44B	0.86853	0.8167	−0.33082	0.0514*	
H45A	0.68295	0.98447	−0.36441	0.0541*	
H45B	0.60843	1.09236	−0.30335	0.0541*	
H46A	0.51838	0.89439	−0.21641	0.0435*	
H46B	0.43828	0.92643	−0.29376	0.0435*	
H47A	0.37225	0.67984	−0.28225	0.0538*	
H47B	0.45203	0.65426	−0.20432	0.0538*	
H48A	0.45417	0.44319	−0.26576	0.0519*	
H48B	0.55108	0.51345	−0.34307	0.0519*	
H49A	0.71053	0.31531	−0.31931	0.0465*	
H49B	0.68437	0.266	−0.22725	0.0465*	
H50A	0.9391	0.22923	−0.26928	0.0481*	
H50B	0.94854	0.38219	−0.31293	0.0481*	
H51A	1.27242	0.77439	−0.05623	0.1031*	
H51B	1.2499	0.62005	−0.0756	0.1031*	
H51C	1.24968	0.65275	0.01334	0.1031*	

### Atomic displacement parameters (Å^2^)

**Table:**

	*U*^11^	*U*^22^	*U*^33^	*U*^12^	*U*^13^	*U*^23^
Co1	0.0355 (9)	0.0233 (7)	0.0266 (8)	−0.0017 (6)	−0.0056 (7)	0.0036 (6)
Cl1	0.048 (2)	0.0461 (18)	0.0415 (17)	0.0059 (16)	−0.0020 (15)	0.0076 (14)
Cl2	0.075 (3)	0.0331 (17)	0.0392 (17)	−0.0001 (16)	−0.0137 (18)	0.0077 (14)
Cl3	0.0405 (17)	0.0311 (14)	0.0323 (14)	−0.0015 (13)	−0.0064 (13)	0.0018 (12)
O1	0.035 (5)	0.039 (5)	0.041 (5)	0.000 (4)	0.009 (4)	0.001 (4)
O2	0.042 (5)	0.032 (4)	0.039 (5)	0.007 (4)	−0.005 (4)	0.006 (4)
O3	0.041 (5)	0.037 (5)	0.043 (5)	−0.006 (4)	−0.006 (4)	−0.011 (4)
O4	0.055 (6)	0.024 (4)	0.034 (4)	0.002 (4)	−0.007 (4)	0.005 (3)
O5	0.056 (6)	0.026 (4)	0.041 (5)	0.001 (4)	−0.012 (4)	−0.003 (4)
O6	0.043 (5)	0.034 (4)	0.030 (4)	−0.005 (4)	−0.010 (4)	0.001 (4)
O7	0.058 (6)	0.030 (4)	0.041 (5)	0.007 (4)	−0.015 (4)	0.000 (4)
O8	0.052 (6)	0.027 (4)	0.053 (5)	−0.012 (4)	−0.015 (5)	0.008 (4)
O9	0.063 (8)	0.120 (11)	0.095 (9)	0.027 (8)	−0.022 (7)	−0.066 (9)
O10	0.21 (2)	0.080 (9)	0.048 (7)	−0.033 (11)	0.028 (10)	0.016 (7)
O11	0.063 (9)	0.083 (10)	0.162 (15)	−0.031 (8)	−0.002 (10)	−0.031 (10)
O12	0.134 (16)	0.101 (12)	0.22 (2)	0.054 (12)	−0.097 (16)	−0.038 (14)
O13	0.063 (7)	0.056 (6)	0.054 (6)	−0.003 (5)	−0.023 (5)	0.034 (5)
O14	0.089 (9)	0.067 (7)	0.031 (5)	−0.026 (6)	−0.013 (5)	0.001 (5)
O15	0.080 (9)	0.070 (8)	0.104 (10)	0.047 (7)	−0.003 (8)	0.012 (7)
O16	0.175 (16)	0.052 (7)	0.051 (7)	−0.047 (8)	0.001 (8)	−0.009 (5)
O17	0.059 (7)	0.057 (6)	0.059 (6)	−0.010 (5)	−0.030 (5)	−0.010 (5)
O18	0.047 (6)	0.036 (5)	0.049 (5)	0.004 (4)	0.005 (5)	0.013 (4)
O19	0.055 (6)	0.045 (5)	0.048 (5)	0.006 (5)	−0.016 (5)	−0.005 (4)
O20	0.047 (6)	0.050 (6)	0.042 (5)	0.000 (5)	0.016 (5)	0.015 (4)
O21	0.063 (7)	0.050 (6)	0.059 (6)	−0.009 (5)	−0.011 (6)	0.012 (5)
O22	0.074 (8)	0.052 (6)	0.044 (6)	0.005 (6)	−0.004 (6)	0.000 (5)
N1	0.033 (6)	0.024 (5)	0.027 (5)	−0.009 (4)	0.003 (4)	0.005 (4)
N2	0.023 (5)	0.029 (5)	0.027 (5)	−0.005 (4)	−0.001 (4)	0.003 (4)
N3	0.032 (5)	0.031 (5)	0.021 (4)	−0.009 (4)	−0.006 (4)	0.004 (4)
N4	0.032 (6)	0.025 (5)	0.032 (5)	−0.005 (4)	−0.004 (4)	0.011 (4)
N5	0.033 (5)	0.025 (5)	0.032 (5)	0.002 (4)	−0.009 (4)	0.003 (4)
N6	0.039 (6)	0.033 (5)	0.021 (5)	−0.008 (4)	−0.013 (4)	0.012 (4)
N7	0.039 (6)	0.025 (5)	0.020 (4)	0.005 (4)	0.001 (4)	0.004 (4)
N8	0.045 (6)	0.027 (5)	0.026 (5)	0.000 (5)	−0.004 (5)	0.012 (4)
C1	0.045 (8)	0.027 (6)	0.031 (6)	−0.002 (5)	−0.008 (6)	0.003 (5)
C2	0.054 (9)	0.027 (6)	0.031 (6)	0.004 (6)	−0.012 (6)	0.006 (5)
C3	0.034 (7)	0.031 (6)	0.043 (7)	−0.001 (5)	−0.003 (6)	0.004 (6)
C4	0.037 (7)	0.031 (6)	0.032 (6)	−0.004 (5)	0.001 (6)	0.003 (5)
C5	0.024 (6)	0.025 (5)	0.031 (6)	0.003 (5)	0.006 (5)	0.003 (5)
C6	0.031 (6)	0.019 (5)	0.035 (6)	0.000 (5)	0.001 (5)	−0.001 (5)
C7	0.035 (7)	0.028 (6)	0.032 (6)	−0.012 (5)	−0.004 (5)	0.011 (5)
C8	0.024 (6)	0.029 (6)	0.029 (6)	−0.003 (5)	−0.001 (5)	0.006 (5)
C9	0.029 (6)	0.027 (5)	0.025 (5)	−0.003 (5)	−0.012 (5)	0.008 (4)
C10	0.036 (7)	0.026 (5)	0.027 (5)	−0.005 (5)	−0.011 (5)	−0.002 (5)
C11	0.042 (7)	0.024 (5)	0.034 (6)	−0.013 (5)	−0.013 (6)	0.004 (5)
C12	0.049 (8)	0.021 (5)	0.026 (5)	0.005 (5)	−0.010 (5)	0.006 (4)
C13	0.039 (7)	0.025 (6)	0.038 (6)	0.006 (5)	−0.011 (6)	−0.001 (5)
C14	0.037 (7)	0.035 (6)	0.029 (6)	−0.012 (6)	−0.005 (5)	0.015 (5)
C15	0.046 (8)	0.029 (6)	0.033 (6)	−0.004 (5)	−0.002 (6)	0.008 (5)
C16	0.037 (7)	0.024 (5)	0.031 (6)	−0.001 (5)	0.007 (5)	−0.002 (5)
C17	0.038 (7)	0.033 (6)	0.038 (6)	−0.009 (5)	−0.007 (6)	0.003 (5)
C18	0.036 (7)	0.033 (6)	0.049 (7)	−0.003 (6)	−0.002 (6)	0.012 (6)
C19	0.041 (8)	0.041 (7)	0.044 (7)	−0.011 (6)	−0.015 (6)	0.009 (6)
C20	0.044 (8)	0.032 (7)	0.056 (8)	−0.006 (6)	−0.005 (7)	0.008 (6)
C21	0.044 (8)	0.030 (6)	0.048 (7)	0.001 (6)	−0.012 (6)	−0.004 (6)
C22	0.055 (9)	0.033 (6)	0.032 (6)	0.002 (6)	−0.005 (6)	−0.008 (5)
C23	0.055 (9)	0.027 (6)	0.040 (7)	0.010 (6)	0.012 (6)	−0.008 (5)
C24	0.053 (8)	0.032 (6)	0.024 (6)	0.002 (6)	−0.005 (6)	0.002 (5)
C25	0.040 (7)	0.024 (5)	0.033 (6)	−0.001 (5)	−0.004 (5)	0.006 (5)
C26	0.037 (7)	0.030 (6)	0.032 (6)	0.000 (5)	0.002 (6)	0.006 (5)
C27	0.042 (8)	0.027 (6)	0.036 (7)	−0.006 (5)	0.000 (6)	0.004 (5)
C28	0.049 (8)	0.033 (6)	0.034 (6)	0.001 (6)	−0.006 (6)	−0.008 (5)
C29	0.038 (7)	0.031 (6)	0.032 (6)	−0.002 (5)	−0.013 (5)	0.009 (5)
C30	0.032 (6)	0.026 (5)	0.023 (5)	0.005 (5)	0.000 (5)	−0.002 (4)
C31	0.043 (7)	0.029 (6)	0.026 (6)	−0.001 (5)	−0.012 (5)	0.007 (5)
C32	0.033 (7)	0.031 (6)	0.033 (6)	−0.002 (5)	−0.004 (5)	0.000 (5)
C33	0.037 (7)	0.030 (6)	0.028 (6)	0.002 (5)	0.006 (5)	0.005 (5)
C34	0.040 (7)	0.027 (5)	0.027 (6)	−0.002 (5)	−0.009 (5)	0.007 (5)
C35	0.035 (7)	0.028 (6)	0.036 (6)	−0.002 (5)	−0.006 (5)	0.006 (5)
C36	0.042 (7)	0.023 (5)	0.026 (5)	0.004 (5)	−0.007 (5)	0.006 (4)
C37	0.041 (7)	0.028 (6)	0.034 (6)	−0.001 (5)	0.004 (6)	0.004 (5)
C38	0.063 (10)	0.024 (6)	0.044 (7)	−0.009 (6)	−0.012 (7)	0.003 (5)
C39	0.048 (8)	0.036 (7)	0.040 (7)	−0.006 (6)	−0.008 (6)	−0.003 (6)
C40	0.043 (7)	0.031 (6)	0.014 (5)	0.002 (5)	0.001 (5)	0.007 (4)
C41	0.056 (8)	0.024 (5)	0.027 (5)	−0.009 (5)	−0.003 (6)	0.007 (5)
C42	0.050 (8)	0.020 (5)	0.049 (7)	−0.008 (5)	−0.010 (7)	0.005 (5)
C43	0.049 (9)	0.034 (7)	0.048 (8)	−0.006 (6)	0.000 (7)	0.007 (6)
C44	0.057 (9)	0.035 (7)	0.035 (7)	−0.006 (6)	−0.006 (6)	0.008 (6)
C45	0.061 (9)	0.031 (6)	0.047 (7)	0.005 (6)	−0.026 (7)	−0.002 (6)
C46	0.044 (7)	0.032 (6)	0.028 (6)	0.007 (5)	0.000 (5)	0.005 (5)
C47	0.045 (8)	0.036 (7)	0.048 (8)	−0.002 (6)	0.002 (7)	0.005 (6)
C48	0.054 (9)	0.036 (7)	0.044 (7)	−0.010 (6)	−0.021 (7)	0.006 (6)
C49	0.055 (9)	0.031 (6)	0.031 (6)	−0.005 (6)	−0.013 (6)	0.007 (5)
C50	0.066 (10)	0.028 (6)	0.028 (6)	−0.004 (6)	−0.013 (6)	0.004 (5)
C51	0.039 (9)	0.096 (13)	0.069 (11)	0.019 (9)	0.004 (8)	−0.027 (10)

### Geometric parameters (Å, º)

**Table:**

Co1—N1	1.910 (10)	C33—C34	1.430 (17)
Co1—N2	1.844 (9)	C34—C35	1.374 (15)
Co1—N3	1.949 (10)	C35—C36	1.457 (17)
Co1—N5	1.940 (10)	C36—C37	1.401 (17)
Co1—N6	1.844 (9)	C37—C38	1.375 (19)
Co1—N7	1.944 (10)	C38—C39	1.334 (18)
Cl1—O9	1.433 (15)	C39—C40	1.409 (19)
Cl1—O10	1.322 (13)	C41—C42	1.529 (17)
Cl1—O11	1.425 (15)	C43—C44	1.53 (2)
Cl1—O12	1.42 (2)	C45—C46	1.478 (19)
Cl2—O13	1.424 (11)	C47—C48	1.485 (18)
Cl2—O14	1.427 (10)	C49—C50	1.49 (2)
Cl2—O15	1.484 (15)	O21—H21	0.820
Cl2—O16	1.444 (15)	O22—H5	0.86 (13)
Cl3—O17	1.435 (12)	O22—H6	0.86 (12)
Cl3—O18	1.426 (10)	C1—H1	0.930
Cl3—O19	1.448 (10)	C2—H2	0.930
Cl3—O20	1.434 (9)	C3—H3	0.930
O1—C17	1.413 (15)	C4—H4	0.930
O1—C18	1.433 (17)	C7—H7	0.930
O2—C19	1.443 (17)	C9—H9	0.930
O2—C20	1.427 (15)	C12—H12	0.930
O3—C21	1.412 (17)	C13—H13	0.930
O3—C22	1.428 (15)	C14—H14	0.930
O4—C23	1.418 (15)	C15—H15	0.930
O4—C24	1.421 (14)	C16—H16A	0.970
O5—C48	1.427 (18)	C16—H16B	0.970
O5—C49	1.435 (14)	C17—H17A	0.970
O6—C46	1.413 (14)	C17—H17B	0.970
O6—C47	1.432 (16)	C18—H18A	0.970
O7—C44	1.418 (16)	C18—H18B	0.970
O7—C45	1.463 (19)	C19—H19A	0.970
O8—C42	1.442 (16)	C19—H19B	0.970
O8—C43	1.431 (15)	C20—H20A	0.970
O21—C51	1.44 (2)	C20—H20B	0.970
N1—C1	1.355 (16)	C21—H21A	0.970
N1—C5	1.382 (14)	C21—H21B	0.970
N2—C6	1.366 (14)	C22—H22A	0.970
N2—C10	1.329 (15)	C22—H22B	0.970
N3—C11	1.350 (14)	C23—H23A	0.970
N3—C15	1.333 (15)	C23—H23B	0.970
N4—C8	1.376 (14)	C24—H24A	0.970
N4—C16	1.466 (15)	C24—H24B	0.970
N4—C25	1.455 (16)	C25—H25A	0.970
N5—C26	1.329 (15)	C25—H25B	0.970
N5—C30	1.386 (14)	C26—H26	0.930
N6—C31	1.352 (16)	C27—H27	0.930
N6—C35	1.371 (15)	C28—H28	0.930
N7—C36	1.375 (13)	C29—H29	0.930
N7—C40	1.346 (14)	C32—H32	0.930
N8—C33	1.345 (15)	C34—H34	0.930
N8—C41	1.468 (16)	C37—H37	0.930
N8—C50	1.469 (17)	C38—H38	0.930
C1—C2	1.410 (19)	C39—H39	0.930
C2—C3	1.383 (17)	C40—H40	0.930
C3—C4	1.375 (18)	C41—H41A	0.970
C4—C5	1.370 (16)	C41—H41B	0.970
C5—C6	1.474 (16)	C42—H42A	0.970
C6—C7	1.374 (15)	C42—H42B	0.970
C7—C8	1.412 (17)	C43—H43A	0.970
C8—C9	1.436 (16)	C43—H43B	0.970
C9—C10	1.393 (14)	C44—H44A	0.970
C10—C11	1.481 (17)	C44—H44B	0.970
C11—C12	1.383 (18)	C45—H45A	0.970
C12—C13	1.383 (17)	C45—H45B	0.970
C13—C14	1.406 (16)	C46—H46A	0.970
C14—C15	1.360 (18)	C46—H46B	0.970
C16—C17	1.533 (18)	C47—H47A	0.970
C18—C19	1.538 (18)	C47—H47B	0.970
C20—C21	1.482 (19)	C48—H48A	0.970
C22—C23	1.52 (2)	C48—H48B	0.970
C24—C25	1.537 (16)	C49—H49A	0.970
C26—C27	1.378 (19)	C49—H49B	0.970
C27—C28	1.412 (17)	C50—H50A	0.970
C28—C29	1.398 (17)	C50—H50B	0.970
C29—C30	1.398 (18)	C51—H51A	0.960
C30—C31	1.478 (16)	C51—H51B	0.960
C31—C32	1.366 (16)	C51—H51C	0.960
C32—C33	1.437 (17)		
			
N1—Co1—N2	82.8 (4)	C4—C3—H3	120.021
N1—Co1—N3	164.5 (4)	C3—C4—H4	120.141
N1—Co1—N5	91.1 (4)	C5—C4—H4	120.134
N1—Co1—N6	98.1 (4)	C6—C7—H7	120.652
N1—Co1—N7	90.5 (4)	C8—C7—H7	120.652
N2—Co1—N3	81.7 (4)	C8—C9—H9	121.141
N2—Co1—N5	99.0 (4)	C10—C9—H9	121.144
N2—Co1—N6	178.3 (5)	C11—C12—H12	119.971
N2—Co1—N7	95.9 (4)	C13—C12—H12	119.965
N3—Co1—N5	91.2 (4)	C12—C13—H13	121.168
N3—Co1—N6	97.5 (4)	C14—C13—H13	121.166
N3—Co1—N7	91.2 (4)	C13—C14—H14	120.481
N5—Co1—N6	82.5 (4)	C15—C14—H14	120.483
N5—Co1—N7	165.2 (4)	N3—C15—H15	118.383
N6—Co1—N7	82.7 (4)	C14—C15—H15	118.390
O9—Cl1—O10	110.7 (9)	N4—C16—H16A	109.257
O9—Cl1—O11	107.3 (8)	N4—C16—H16B	109.253
O9—Cl1—O12	107.7 (11)	C17—C16—H16A	109.253
O10—Cl1—O11	111.5 (12)	C17—C16—H16B	109.259
O10—Cl1—O12	111.0 (13)	H16A—C16—H16B	107.926
O11—Cl1—O12	108.5 (10)	O1—C17—H17A	110.452
O13—Cl2—O14	110.7 (6)	O1—C17—H17B	110.444
O13—Cl2—O15	108.7 (7)	C16—C17—H17A	110.452
O13—Cl2—O16	111.3 (8)	C16—C17—H17B	110.448
O14—Cl2—O15	108.3 (8)	H17A—C17—H17B	108.644
O14—Cl2—O16	107.4 (7)	O1—C18—H18A	108.759
O15—Cl2—O16	110.4 (9)	O1—C18—H18B	108.762
O17—Cl3—O18	108.1 (6)	C19—C18—H18A	108.763
O17—Cl3—O19	108.1 (6)	C19—C18—H18B	108.766
O17—Cl3—O20	111.7 (6)	H18A—C18—H18B	107.654
O18—Cl3—O19	110.5 (6)	O2—C19—H19A	110.294
O18—Cl3—O20	108.9 (6)	O2—C19—H19B	110.295
O19—Cl3—O20	109.5 (6)	C18—C19—H19A	110.291
C17—O1—C18	111.2 (10)	C18—C19—H19B	110.289
C19—O2—C20	110.2 (10)	H19A—C19—H19B	108.538
C21—O3—C22	111.7 (10)	O2—C20—H20A	108.474
C23—O4—C24	112.8 (9)	O2—C20—H20B	108.478
C48—O5—C49	112.6 (10)	C21—C20—H20A	108.465
C46—O6—C47	113.5 (9)	C21—C20—H20B	108.478
C44—O7—C45	110.5 (10)	H20A—C20—H20B	107.499
C42—O8—C43	114.5 (11)	O3—C21—H21A	109.410
Co1—N1—C1	127.0 (8)	O3—C21—H21B	109.415
Co1—N1—C5	115.2 (7)	C20—C21—H21A	109.416
C1—N1—C5	117.8 (10)	C20—C21—H21B	109.416
Co1—N2—C6	118.8 (7)	H21A—C21—H21B	108.037
Co1—N2—C10	120.1 (7)	O3—C22—H22A	109.885
C6—N2—C10	121.0 (9)	O3—C22—H22B	109.885
Co1—N3—C11	114.3 (8)	C23—C22—H22A	109.877
Co1—N3—C15	126.9 (8)	C23—C22—H22B	109.879
C11—N3—C15	118.8 (11)	H22A—C22—H22B	108.297
C8—N4—C16	122.0 (10)	O4—C23—H23A	109.006
C8—N4—C25	122.2 (9)	O4—C23—H23B	109.008
C16—N4—C25	115.1 (9)	C22—C23—H23A	109.009
Co1—N5—C26	127.4 (8)	C22—C23—H23B	109.015
Co1—N5—C30	113.6 (7)	H23A—C23—H23B	107.796
C26—N5—C30	118.8 (10)	O4—C24—H24A	110.387
Co1—N6—C31	120.2 (8)	O4—C24—H24B	110.388
Co1—N6—C35	119.1 (8)	C25—C24—H24A	110.389
C31—N6—C35	120.7 (9)	C25—C24—H24B	110.393
Co1—N7—C36	113.4 (7)	H24A—C24—H24B	108.597
Co1—N7—C40	127.0 (7)	N4—C25—H25A	108.460
C36—N7—C40	119.6 (10)	N4—C25—H25B	108.456
C33—N8—C41	120.0 (11)	C24—C25—H25A	108.463
C33—N8—C50	121.3 (11)	C24—C25—H25B	108.461
C41—N8—C50	118.4 (9)	H25A—C25—H25B	107.486
N1—C1—C2	122.0 (10)	N5—C26—H26	118.708
C1—C2—C3	118.3 (12)	C27—C26—H26	118.704
C2—C3—C4	120.0 (12)	C26—C27—H27	120.236
C3—C4—C5	119.7 (11)	C28—C27—H27	120.239
N1—C5—C4	122.0 (10)	C27—C28—H28	120.568
N1—C5—C6	112.1 (9)	C29—C28—H28	120.559
C4—C5—C6	125.9 (10)	C28—C29—H29	120.888
N2—C6—C5	111.0 (9)	C30—C29—H29	120.881
N2—C6—C7	121.6 (11)	C31—C32—H32	120.008
C5—C6—C7	127.4 (11)	C33—C32—H32	120.011
C6—C7—C8	118.7 (11)	C33—C34—H34	119.888
N4—C8—C7	122.9 (10)	C35—C34—H34	119.902
N4—C8—C9	118.0 (10)	C36—C37—H37	120.610
C7—C8—C9	119.0 (10)	C38—C37—H37	120.594
C8—C9—C10	117.7 (10)	C37—C38—H38	119.558
N2—C10—C9	121.9 (10)	C39—C38—H38	119.575
N2—C10—C11	110.9 (9)	C38—C39—H39	119.913
C9—C10—C11	127.1 (11)	C40—C39—H39	119.926
N3—C11—C10	113.0 (11)	N7—C40—H40	119.898
N3—C11—C12	121.2 (11)	C39—C40—H40	119.886
C10—C11—C12	125.8 (10)	N8—C41—H41A	109.396
C11—C12—C13	120.1 (10)	N8—C41—H41B	109.392
C12—C13—C14	117.7 (11)	C42—C41—H41A	109.398
C13—C14—C15	119.0 (11)	C42—C41—H41B	109.394
N3—C15—C14	123.2 (11)	H41A—C41—H41B	108.007
N4—C16—C17	111.8 (9)	O8—C42—H42A	109.132
O1—C17—C16	106.4 (10)	O8—C42—H42B	109.132
O1—C18—C19	114.0 (11)	C41—C42—H42A	109.128
O2—C19—C18	107.1 (11)	C41—C42—H42B	109.134
O2—C20—C21	115.2 (11)	H42A—C42—H42B	107.861
O3—C21—C20	111.1 (11)	O8—C43—H43A	108.294
O3—C22—C23	109.0 (11)	O8—C43—H43B	108.291
O4—C23—C22	112.9 (11)	C44—C43—H43A	108.307
O4—C24—C25	106.7 (9)	C44—C43—H43B	108.303
N4—C25—C24	115.3 (11)	H43A—C43—H43B	107.409
N5—C26—C27	122.6 (11)	O7—C44—H44A	109.754
C26—C27—C28	119.5 (11)	O7—C44—H44B	109.758
C27—C28—C29	118.9 (12)	C43—C44—H44A	109.758
C28—C29—C30	118.2 (11)	C43—C44—H44B	109.758
N5—C30—C29	121.9 (10)	H44A—C44—H44B	108.224
N5—C30—C31	113.3 (10)	O7—C45—H45A	109.466
C29—C30—C31	124.8 (10)	O7—C45—H45B	109.463
N6—C31—C30	110.3 (9)	C46—C45—H45A	109.470
N6—C31—C32	121.7 (11)	C46—C45—H45B	109.471
C30—C31—C32	128.0 (11)	H45A—C45—H45B	108.057
C31—C32—C33	120.0 (12)	O6—C46—H46A	109.215
N8—C33—C32	121.8 (11)	O6—C46—H46B	109.219
N8—C33—C34	121.5 (11)	C45—C46—H46A	109.216
C32—C33—C34	116.7 (10)	C45—C46—H46B	109.217
C33—C34—C35	120.2 (11)	H46A—C46—H46B	107.910
N6—C35—C34	120.7 (11)	O6—C47—H47A	109.862
N6—C35—C36	110.5 (9)	O6—C47—H47B	109.865
C34—C35—C36	128.7 (11)	C48—C47—H47A	109.870
N7—C36—C35	114.2 (10)	C48—C47—H47B	109.868
N7—C36—C37	120.3 (10)	H47A—C47—H47B	108.282
C35—C36—C37	125.5 (10)	O5—C48—H48A	110.013
C36—C37—C38	118.8 (11)	O5—C48—H48B	110.017
C37—C38—C39	120.9 (13)	C47—C48—H48A	110.013
C38—C39—C40	120.2 (13)	C47—C48—H48B	110.016
N7—C40—C39	120.2 (10)	H48A—C48—H48B	108.374
N8—C41—C42	111.2 (11)	O5—C49—H49A	109.631
O8—C42—C41	112.4 (11)	O5—C49—H49B	109.634
O8—C43—C44	115.9 (12)	C50—C49—H49A	109.629
O7—C44—C43	109.6 (11)	C50—C49—H49B	109.629
O7—C45—C46	110.9 (11)	H49A—C49—H49B	108.155
O6—C46—C45	112.0 (10)	N8—C50—H50A	108.683
O6—C47—C48	109.1 (11)	N8—C50—H50B	108.676
O5—C48—C47	108.4 (12)	C49—C50—H50A	108.681
O5—C49—C50	110.1 (11)	C49—C50—H50B	108.680
N8—C50—C49	114.3 (10)	H50A—C50—H50B	107.616
C51—O21—H21	109.475	O21—C51—H51A	109.476
H5—O22—H6	107 (16)	O21—C51—H51B	109.467
N1—C1—H1	119.021	O21—C51—H51C	109.476
C2—C1—H1	119.019	H51A—C51—H51B	109.467
C1—C2—H2	120.833	H51A—C51—H51C	109.471
C3—C2—H2	120.842	H51B—C51—H51C	109.469
C2—C3—H3	120.023		
			
N1—Co1—N2—C6	−1.5 (7)	C25—N4—C16—C17	−79.3 (12)
N1—Co1—N2—C10	179.7 (8)	Co1—N5—C26—C27	175.0 (7)
N2—Co1—N1—C1	−178.0 (8)	Co1—N5—C30—C29	−176.7 (7)
N2—Co1—N1—C5	3.0 (6)	Co1—N5—C30—C31	3.3 (11)
N1—Co1—N5—C26	−80.1 (8)	C26—N5—C30—C29	−0.1 (16)
N1—Co1—N5—C30	96.2 (6)	C26—N5—C30—C31	179.9 (9)
N5—Co1—N1—C1	−79.1 (8)	C30—N5—C26—C27	−1.1 (17)
N5—Co1—N1—C5	101.9 (6)	Co1—N6—C31—C30	1.9 (13)
N1—Co1—N6—C31	−90.3 (8)	Co1—N6—C31—C32	−178.2 (7)
N1—Co1—N6—C35	92.1 (8)	Co1—N6—C35—C34	177.2 (7)
N6—Co1—N1—C1	3.5 (9)	Co1—N6—C35—C36	−3.7 (13)
N6—Co1—N1—C5	−175.5 (6)	C31—N6—C35—C34	−0.5 (17)
N1—Co1—N7—C36	−98.9 (6)	C31—N6—C35—C36	178.6 (10)
N1—Co1—N7—C40	78.2 (8)	C35—N6—C31—C30	179.6 (9)
N7—Co1—N1—C1	86.2 (8)	C35—N6—C31—C32	−0.5 (18)
N7—Co1—N1—C5	−92.9 (6)	Co1—N7—C36—C35	−1.0 (12)
N2—Co1—N3—C11	−0.3 (6)	Co1—N7—C36—C37	179.2 (7)
N2—Co1—N3—C15	−179.8 (9)	Co1—N7—C40—C39	−179.8 (7)
N3—Co1—N2—C6	178.5 (8)	C36—N7—C40—C39	−2.9 (16)
N3—Co1—N2—C10	−0.3 (7)	C40—N7—C36—C35	−178.2 (9)
N2—Co1—N5—C26	2.8 (9)	C40—N7—C36—C37	1.9 (16)
N2—Co1—N5—C30	179.1 (6)	C33—N8—C41—C42	−86.0 (13)
N5—Co1—N2—C6	−91.6 (7)	C41—N8—C33—C32	−172.5 (10)
N5—Co1—N2—C10	89.7 (8)	C41—N8—C33—C34	6.7 (17)
N2—Co1—N7—C36	178.3 (6)	C33—N8—C50—C49	63.4 (14)
N2—Co1—N7—C40	−4.6 (8)	C50—N8—C33—C32	13.5 (17)
N7—Co1—N2—C6	88.2 (7)	C50—N8—C33—C34	−167.3 (10)
N7—Co1—N2—C10	−90.6 (7)	C41—N8—C50—C49	−110.7 (11)
N3—Co1—N5—C26	84.6 (8)	C50—N8—C41—C42	88.1 (12)
N3—Co1—N5—C30	−99.2 (6)	N1—C1—C2—C3	2.2 (18)
N5—Co1—N3—C11	−99.2 (7)	C1—C2—C3—C4	−5.1 (19)
N5—Co1—N3—C15	81.3 (8)	C2—C3—C4—C5	4.4 (19)
N3—Co1—N6—C31	90.1 (8)	C3—C4—C5—N1	−0.6 (18)
N3—Co1—N6—C35	−87.6 (7)	C3—C4—C5—C6	−180.0 (11)
N6—Co1—N3—C11	178.2 (6)	N1—C5—C6—N2	2.5 (13)
N6—Co1—N3—C15	−1.3 (9)	N1—C5—C6—C7	−180.0 (10)
N3—Co1—N7—C36	96.6 (6)	C4—C5—C6—N2	−178.1 (11)
N3—Co1—N7—C40	−86.4 (8)	C4—C5—C6—C7	−0.6 (19)
N7—Co1—N3—C11	95.5 (6)	N2—C6—C7—C8	−1.5 (17)
N7—Co1—N3—C15	−84.1 (8)	C5—C6—C7—C8	−178.9 (10)
N5—Co1—N6—C31	−0.2 (7)	C6—C7—C8—N4	178.6 (10)
N5—Co1—N6—C35	−177.9 (8)	C6—C7—C8—C9	2.6 (17)
N6—Co1—N5—C26	−178.1 (9)	N4—C8—C9—C10	−179.2 (9)
N6—Co1—N5—C30	−1.8 (6)	C7—C8—C9—C10	−3.1 (16)
N6—Co1—N7—C36	−0.8 (6)	C8—C9—C10—N2	2.6 (17)
N6—Co1—N7—C40	176.2 (8)	C8—C9—C10—C11	178.5 (10)
N7—Co1—N6—C31	−179.7 (8)	N2—C10—C11—N3	−0.9 (14)
N7—Co1—N6—C35	2.6 (7)	N2—C10—C11—C12	−179.6 (10)
C17—O1—C18—C19	55.3 (12)	C9—C10—C11—N3	−177.2 (11)
C18—O1—C17—C16	164.1 (8)	C9—C10—C11—C12	4 (2)
C19—O2—C20—C21	66.0 (14)	N3—C11—C12—C13	0.7 (18)
C20—O2—C19—C18	174.2 (9)	C10—C11—C12—C13	179.3 (10)
C21—O3—C22—C23	−175.3 (8)	C11—C12—C13—C14	−0.8 (17)
C22—O3—C21—C20	−167.2 (8)	C12—C13—C14—C15	1.1 (18)
C23—O4—C24—C25	176.6 (9)	C13—C14—C15—N3	−1.4 (19)
C24—O4—C23—C22	−74.7 (12)	N4—C16—C17—O1	170.5 (8)
C48—O5—C49—C50	153.2 (9)	O1—C18—C19—O2	55.2 (13)
C49—O5—C48—C47	177.9 (8)	O2—C20—C21—O3	60.1 (15)
C46—O6—C47—C48	−177.6 (9)	O3—C22—C23—O4	−53.7 (12)
C47—O6—C46—C45	178.5 (9)	O4—C24—C25—N4	−142.6 (9)
C44—O7—C45—C46	101.9 (11)	N5—C26—C27—C28	1.0 (18)
C45—O7—C44—C43	−168.2 (9)	C26—C27—C28—C29	0.4 (18)
C42—O8—C43—C44	57.6 (13)	C27—C28—C29—C30	−1.5 (18)
C43—O8—C42—C41	91.5 (12)	C28—C29—C30—N5	1.4 (17)
Co1—N1—C1—C2	−177.5 (7)	C28—C29—C30—C31	−178.6 (10)
Co1—N1—C5—C4	176.8 (7)	N5—C30—C31—N6	−3.3 (14)
Co1—N1—C5—C6	−3.7 (11)	N5—C30—C31—C32	176.8 (10)
C1—N1—C5—C4	−2.3 (16)	C29—C30—C31—N6	176.7 (10)
C1—N1—C5—C6	177.2 (9)	C29—C30—C31—C32	−3 (2)
C5—N1—C1—C2	1.5 (16)	N6—C31—C32—C33	0.4 (18)
Co1—N2—C6—C5	−0.1 (12)	C30—C31—C32—C33	−179.8 (10)
Co1—N2—C6—C7	−177.8 (7)	C31—C32—C33—N8	−179.9 (11)
Co1—N2—C10—C9	177.3 (7)	C31—C32—C33—C34	0.8 (17)
Co1—N2—C10—C11	0.7 (13)	N8—C33—C34—C35	178.9 (10)
C6—N2—C10—C9	−1.5 (17)	C32—C33—C34—C35	−1.8 (17)
C6—N2—C10—C11	−178.0 (9)	C33—C34—C35—N6	1.7 (18)
C10—N2—C6—C5	178.7 (9)	C33—C34—C35—C36	−177.3 (10)
C10—N2—C6—C7	0.9 (17)	N6—C35—C36—N7	2.8 (14)
Co1—N3—C11—C10	0.7 (12)	N6—C35—C36—C37	−177.3 (10)
Co1—N3—C11—C12	179.5 (7)	C34—C35—C36—N7	−178.1 (11)
Co1—N3—C15—C14	−179.2 (7)	C34—C35—C36—C37	2 (2)
C11—N3—C15—C14	1.3 (18)	N7—C36—C37—C38	−0.7 (17)
C15—N3—C11—C10	−179.7 (10)	C35—C36—C37—C38	179.4 (10)
C15—N3—C11—C12	−0.9 (17)	C36—C37—C38—C39	1 (2)
C8—N4—C16—C17	91.8 (11)	C37—C38—C39—C40	−2 (2)
C16—N4—C8—C7	6.8 (17)	C38—C39—C40—N7	3 (2)
C16—N4—C8—C9	−177.1 (9)	N8—C41—C42—O8	167.0 (9)
C8—N4—C25—C24	−68.2 (14)	O8—C43—C44—O7	55.0 (14)
C25—N4—C8—C7	177.3 (10)	O7—C45—C46—O6	−72.7 (13)
C25—N4—C8—C9	−6.7 (16)	O6—C47—C48—O5	63.1 (13)
C16—N4—C25—C24	102.8 (10)	O5—C49—C50—N8	50.2 (12)

### Hydrogen-bond geometry (Å, º)

*Cg*1 and *Cg*2 are the centroids of the N7/C36–C40 and N2/C6–C10 pyridine rings, respectively.

**Table:**

*D*—H···*A*	*D*—H	H···*A*	*D*···*A*	*D*—H···*A*
O21—H21···O8	0.82	1.99	2.802 (15)	173
O22—H5···O9	0.86 (13)	2.11 (14)	2.960 (19)	172 (15)
O22—H6···O5	0.86 (12)	2.17 (11)	2.934 (13)	148 (14)
C4—H4···O18	0.93	2.56	3.299 (16)	137
C15—H15···O11^i^	0.93	2.54	3.35 (2)	145
C16—H16*B*···O6^ii^	0.97	2.58	3.491 (15)	157
C20—H20*B*···O10^ii^	0.97	2.54	3.50 (2)	170
C22—H22*A*···O13^iii^	0.97	2.41	3.365 (17)	168
C25—H25*A*···O6^ii^	0.97	2.59	3.517 (14)	160
C25—H25*B*···O20^i^	0.97	2.49	3.331 (15)	145
C26—H26···O16^iii^	0.93	2.54	3.400 (17)	155
C27—H27···O14^iii^	0.93	2.45	3.249 (16)	143
C28—H28···O22^iii^	0.93	2.47	3.290 (17)	147
C29—H29···O7^iii^	0.93	2.51	3.437 (14)	175
C37—H37···O21	0.93	2.35	3.276 (16)	177
C44—H44*A*···O20^iv^	0.97	2.49	3.297 (17)	141
C3—H3···*Cg*1^v^	0.93	2.70	3.540 (15)	151
C40—H40···*Cg*2	0.93	2.97	3.713 (12)	138

Symmetry codes: (i) *x*+1, *y*, *z*; (ii) *x*, *y*−1, *z*+1; (iii) *x*, *y*−1, *z*; (iv) *x*+1, *y*+1, *z*−1; (v) *x*−1, *y*, *z*.

## References

[bb1] Burla, M. C., Caliandro, R., Camalli, M., Carrozzini, B., Cascarano, G. L., De Caro, L., Giacovazzo, C., Polidori, G. & Spagna, R. (2005). *J. Appl. Cryst.* **38**, 381–388.

[bb2] Hayami, S., Komatsu, Y., Shimizu, T., Kamihata, H. & Lee, Y. H. (2011). *Coord. Chem. Rev.* **255**, 1981–1990.

[bb3] Parsons, S. & Flack, H. (2004). *Acta Cryst.* A**60**, s61.

[bb4] Rigaku (1995). *RAPID-AUTO* and *ABSCOR*. Rigaku Corporation, Tokyo, Japan.

[bb5] Rigaku (2014). *CrystalStructure*. Rigaku Corporation, Tokyo, Japan.

[bb6] Sheldrick, G. M. (2015). *Acta Cryst.* C**71**, 3–8.

